# Intramural Esophageal Abscess Complicated with Pleural Fistula: A Case Report

**DOI:** 10.7759/cureus.6846

**Published:** 2020-02-02

**Authors:** Shruti Kumar, Muthu Kumar Sakthivel, Thangavijayan Bosemani

**Affiliations:** 1 Radiology, Shri Sai Hospital, Patna, IND; 2 Radiology, University of North Carolina School of Medicine, Chapel Hill, USA

**Keywords:** intramural esophageal abscess, intramural esophageal dissection, pleural fistula, contrast esophagography, computed tomography, double barreled esophagus

## Abstract

Intramural esophageal abscess is a rare entity caused by mucosal injury to the esophagus but without transmural perforation. The mucosal disruption provides access to the intraluminal infectious contents to traverse into the loose submucosal tissue, resulting in an intramural abscess. It is important to be well-versed in the clinical and imaging findings of this pathology in order to make a timely diagnosis. Here, we present a case of intramural esophageal abscess complicated with a pleural fistula with a focus on the radiological features of this rare entity. To our knowledge, this is the first time that an esophageal intramural abscess complicated with pleural fistula is discussed in peer-reviewed literature.

## Introduction

Intramural esophageal abscess is a rare pathology caused by the longitudinal separation of the mucosal and submucosal layers of the esophagus, but without perforation [1-2]. Damage to the esophageal wall may involve only the mucosa, leaving the muscle layer intact. The mucosal laceration may allow access to the infected material to travel from the lumen of the esophagus to enter the loose submucosal layer, producing a longitudinal dissection with separation of the mucosa from the surrounding esophageal muscle [3]. Intramural esophageal dissections are most often iatrogenic manifestations, which follow instrumentation, treatment of varices, and anticoagulation [4]. Common symptoms include retrosternal chest pain, dysphagia, odynophagia, and hematemesis [5]. A prompt diagnosis is of utmost importance. In this report, we present a case of intramural esophageal abscess complicated with a pleural fistula with a focus on the radiological features of this rare entity. To the best of our knowledge, it is the first case of its kind in the literature.

## Case presentation

**Figure 1:**
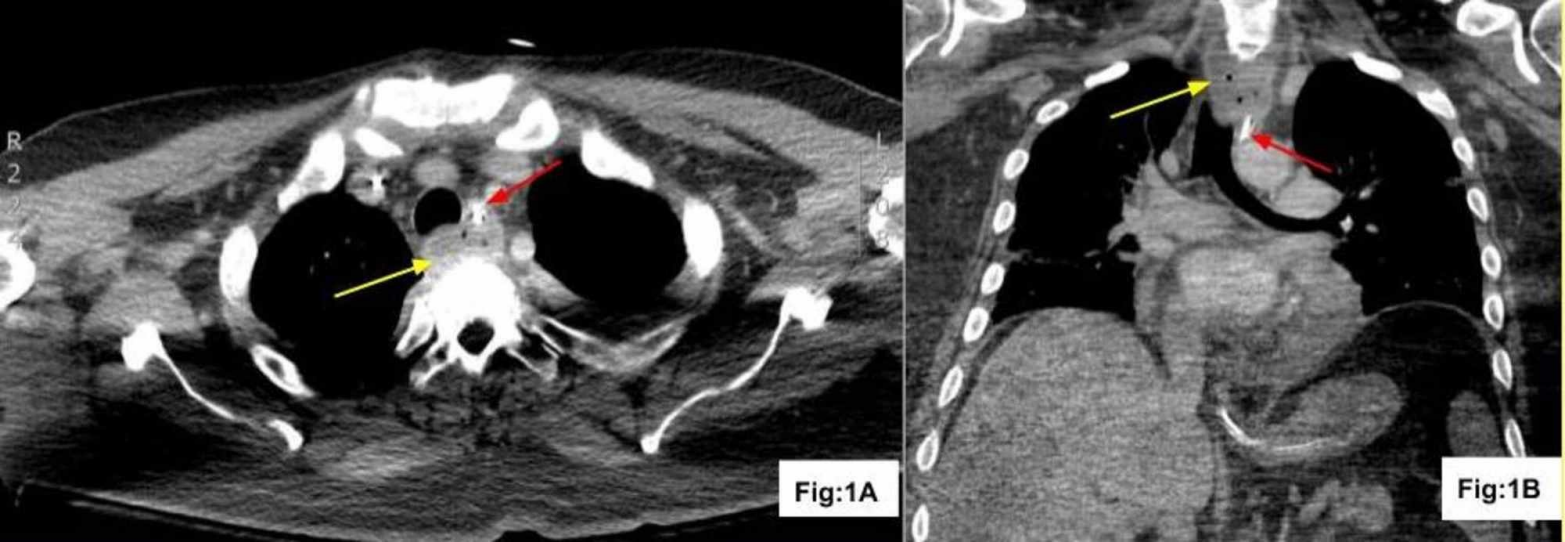
CT of the chest after vancomycin IV treatment CT with contrast axial (Fig: 1A) and coronal (Fig: 1B) reconstruction demonstrates intramural esophageal abscess with tiny gas locules (yellow arrow) displacing the enteric tube anteriorly (red arrow) CT: computed tomography; IV: intravenous

A 42-year-old man presented to the emergency department with multiple complaints that he had been having for nine days, including generalized fatigue, diffuse arthralgia, muscle cramps, and dark urine. The patient reported a medical history of spine surgery treated with neurostimulator placement. On examination, the patient was febrile and tachycardic, with elevated WBC, creatinine, and lactate, complicated by acute renal failure and coagulopathy. The provisional clinical diagnosis was sepsis of unknown etiology. A chest radiograph was unremarkable. A chest CT revealed multiple cavitating pulmonary nodules consistent with septic embolism. Echocardiogram showed an 8x4- mm mobile echogenicity on the ventricular side of the aortic valve along the right coronary cusp, which was consistent with infective endocarditis. The patient was treated with vancomycin IV after methicillin-resistant *Staphylococcus aureus* (MRSA) was identified as the cause for sepsis. A repeat chest CT after two days identified worsening septic emboli and a new small intramural proximal esophageal collection at the level of the thoracic inlet (Figure 1). 

**Figure 2:**
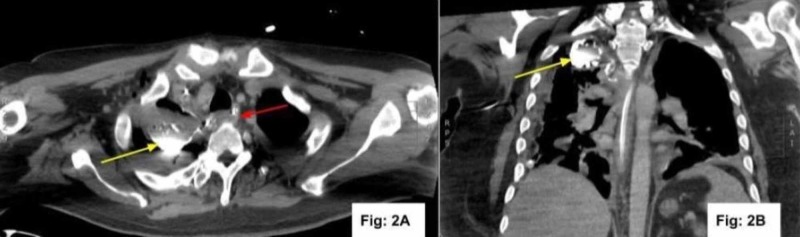
CT of the chest after incision and drainage of the abscess CT with contrast axial (Fig: 2A) and coronal (Fig: 2B) reconstruction demonstrates orally administered barium contrast in the right apical pleural space (yellow arrow) and decrease in size of intramural esophageal abscess (red arrow) consistent with esophageal pleural fistula CT: computed tomography

The patient underwent surgical debridement of vegetation and aortic valve replacement. A chest CT performed on postoperative day 12 revealed similar-looking intramural esophageal collection, septic emboli, and postoperative changes of aortic valve replacement. Postoperative aortic valve pathology showed valvular tissue with acute inflammation and fibrinoid necrosis with associated necroinflammatory vegetations and bacterial colonies. The patient was taken to the operating room and an esophagoscopy was performed, which showed intact esophageal mucosa. The incision and drainage of intramural oesophageal abscess were performed. On the third day following the incision and drainage of the abscess, the patient underwent repeat CT with oral contrast, which demonstrated right-sided empyema with a collection of barium in the right lung apex consistent with esophageal-pleural fistula (Figure 2).

After two days, the patient was again taken to the operating room, and an esophagoscopy showed intact mucosa and no obvious communication between the esophagus and pleural space, which implied possible sealed-off perforation.

## Discussion

Intramural esophageal abscess is a rare pathology and occurs as a consequence of the intermediate level of mucosal injury as opposed to complete esophageal perforation [3]. Mucosal damage may allow the infected contents to leak from the esophageal lumen into the intramural space, thus leading to the formation of intramural abscess [3]. The mechanism of intramural esophageal dissection is the sudden increase in the intraluminal pressure of the esophagus, which is not severe enough to cause a perforation [1]. The clinical features of this entity are also milder compared to complete rupture, and patients usually present with retrosternal pain, hematemesis, odynophagia, and dysphagia [5]. This disease has predominantly been reported in elderly females. However, few case studies have also reported its occurrence in younger male patients [1,6-7]. Predisposing factors include coagulation defects, endoscopic instrumentation, and injection sclerotherapy for esophageal varices [1]. Benatta et al. have described the case of an intramural esophageal dissection without abscess, secondary to pharyngitis [1]. Another case report by Amiraraghi et al. describes the occurrence of intramural esophageal abscess as an unusual complication of tonsillitis [4]. Lichter et al. have described two patients with intramural esophageal abscess formation secondary to iatrogenic trauma [3].

More often, the dissection is partial rather than circumferential, with only two circumferential cases described so far in the literature [1-2]. In the early stages, radiographs of the chest and neck usually appear unremarkable [3]. No evidence of surgical emphysema is evident on plain radiographs of the chest and neck unlike in patients with complete perforation [3]. The diagnosis is usually made by contrast esophagography, esophageal endoscopy, or cross-sectional imaging like CT scan [1]. It may present as a diagnostic dilemma in many cases owing to its rare occurrence and non-specific imaging features. A contrast esophagogram using water-soluble contrast may help to diagnose intramural esophageal dissection, with a demonstration of the characteristic radiological appearance of a “double-barreled esophagus” as a result of the formation of two lumens [3]. Unlike congenital duplication where the communication occurs at the lower end of the “double-barrel”, the communication in iatrogenic intramural esophageal dissection is seen at the upper end [3]. Endoscopy may aid in the diagnosis with a demonstration of two lumens. A CT chest may demonstrate a characteristic “double-barreled esophagus” with the presence of true and false lumens and a mucosal flap suggestive of esophageal dissection. This characteristic finding may not be seen in all patients of intramural esophageal abscesses, and some patients may show the presence of a hypodense collection in the para-esophageal location with possible internal air lucencies and adjacent fat stranding. Other differentials for this imaging finding may include secondarily infected esophageal duplication cyst, neurenteric cyst, lymphatic malformation, and pancreatic pseudocyst with intrathoracic extension. Any complications associated with intramural esophageal abscesses such as fistulous communication with pleura and subsequent pleural collection may also be well-appreciated on CT. Although CT imaging features may be similar to those of other mediastinal pathologies, clinical history, anatomical position, and other pertinent findings such as persistent anterior displacement of the enteric tube on two consecutive studies and absence of an enhancing wall outside the esophagus point towards a primary esophageal pathology in our case report.

## Conclusions

Intramural esophageal abscess is a rare entity that must be considered in appropriate clinical setting, especially in elderly women post-endoscopic procedures and with coagulation abnormalities. It is important for radiologists to understand the clinical and radiological findings of this entity and the possibility of associated complications. Although it remains a diagnostic dilemma in a few cases, cross-sectional imaging with CT may be utilized in order to obtain a correct diagnosis.
